# Follicular Fluid Metabolite Changes in Dairy Cows with Inactive Ovary Identified Using Untargeted Metabolomics

**DOI:** 10.1155/2020/9837543

**Published:** 2020-11-16

**Authors:** YunLong Bai, Feng Zhang, HongYou Zhang, Chuang Xu, Ling Wu, Cheng Xia

**Affiliations:** ^1^College of Animal Science and Veterinary Medicine, Heilongjiang Bayi Agricultural University, Heilongjiang 163319, China; ^2^Heilongjiang Provincial Technology Innovation Center for Bovine Disease Control and Prevention, Daqing 163319, China

## Abstract

The metabolism of dairy cows with inactive ovaries differs from that of healthy dairy cows. However, the molecular mechanisms underpinning these physiological and metabolic changes remain unclear. The purpose of this study was to investigate follicular fluid metabolite changes in dairy cows with inactive ovaries. Untargeted metabolomics technology and multivariate statistical analysis were used to screen differential metabolites in follicular fluid samples between inactive ovaries and estrus cows at 45-60 d postpartum. Fourteen differential metabolites were identified, consisting of amino acids, lipids, sugars, and nucleotides. When compared with healthy animal samples, eight follicular fluid metabolites were significantly increased, and six metabolites were significantly decreased in dairy cows with inactive ovaries. Metabolic pathway analyses indicated that differential metabolites were primarily involved in glycerol phospholipid metabolism, arachidonic acid metabolism, valine, leucine and isoleucine biosynthesis, and phenylalanine metabolism. These metabolites and their enrichment pathways indicate that the enhancement of lipid metabolism and the weakening of carbohydrate production of amino acids in dairy cows with impaired follicular development. Overall, these data provide a better understanding of the changes that could affect follicular development during the postpartum period and lay the ground for further investigations.

## 1. Introduction

For dairy cows, the transition period from late pregnancy to early lactation generally refers to 21 days before to 21 days after calving [1]. During this period, significant physiological changes occur, encompassing nutritional and metabolic alterations. In the late pregnancy period, dry matter intake decreases which may be caused by the physical obstruction of the rumen by the fetus, with simultaneous increases in reproductive hormone levels. Similarly, milk synthesis and secretion postpartum require large quantities of glucose. Therefore, energy consumption is higher than intake, resulting in a negative energy balance [2].

Negative energy balance (NEB) is a serious metabolic disorder in milk high-producing dairy cows, especially during early lactation. When negative energy balance (NEB) occurs in dairy cows, fat mobilization from adipose tissue is rapidly increased; a large amount of fat decomposition increases the content of nonesterified fatty acids (NEFA) and *β*-hydroxybutyrate (BHBA) in the blood. High concentrations of NEFA and BHBA could inhibit the secretion of estrogen (E_2_) and insulin-like growth factor-1 (IGF-1) in plasma [3]. However, both E_2_ and IGF-1 could stimulate the development of follicular cells in dairy cows [4]. Therefore, the dairy cows with NEB postpartum have a higher anestrus rate.

In the past few decades, the annual yield of dairy cows around the world has been greatly increased; however, the reproductive efficiency of dairy cows has gradually decreased [5]. It directly leads to serious damage to the economic benefits of the dairy farm. So far, some techniques, such as synchronous estrus, superovulation, and regular insemination, can improve the reproductive performance of dairy cows, but the problem of low reproductive efficiency has not been well solved [6].

Recent research reports and our previous research showed that anestrus and inactive ovaries are primary factors leading to a low efficiency in postpartum dairy cow reproduction [7]. However, these issues are unrelated to nutrition, metabolism, endocrine, and other mechanisms in the perinatal and early lactation period [8, 9]. The incidence of inactive ovaries in postpartum anestrus in dairy cows is approximately 26.3%, with some incidences as high as 40% and more [10]. In view of the fact that inactive ovaries are becoming more common in postpartum dairy cows with a negative energy balance, the metabolic relationship between the two is still unclear. Thus, further studies in this area are required. Additionally, it is important to identify mechanisms behind postpartum anestrus in these animals.

Metabolomics was first introduced by Nicholson et al. [11]. Targeted metabolomics quantitatively detects metabolites in metabolic pathways, whereas untargeted metabolomics examines metabolite differences between control and experimental groups. Both methods are important in screening for disease markers [12]. In this study, we used untargeted metabolomics to analyze the follicular fluid metabolic spectrum in dairy cows with inactive ovaries. The identification of differential metabolites will help clarify mechanisms in postpartum dairy cows with inactive ovaries.

In view of the fact that the mechanisms behind inactive ovaries are unclear, only limited measures can be used to alleviate the negative effect on the reproductive performance of dairy cows. We used an untargeted metabolomics approach to identify follicular fluid metabolite changes in dairy cows with inactive ovaries. This analysis provides novel information that can be used to elucidate the mechanisms behind this harmful condition.

## 2. Materials and Methods

### 2.1. Animals

Thirty healthy Holstein dairy cows with similar milk yields, parity, body condition score (BCS) at 14 to 21 d were selected for this experiment in an intensive dairy farm of Heilongjiang Province, China. All experimental cows were fed ad libitum with total mixed rations (TMR) that were consisted of cottonseed (1.03 kg), soybean skin (1.5 kg), oat grass (0.5 kg), alfalfa (2.50 kg), soybean meal (1.3 kg), pressed corn (2 kg), molasses (1 kg), silage (25.37 kg), and corn (3 kg).

Blood samples of all experimental cows were collected at 14 to 21 d and 45 to 60 d postpartum by the tail vein to determine the status of energy balance. All experimental cows were tracked to detect follicular size at 45 to 60 d postpartum by B-ultrasound and rectal examination. According to the follicle size and the results of rectal examination at 45 to 60 d postpartum, these cows were divided into estrus (E, the diameter of follicle is more than 8 mm) group and inactive ovaries (IO, the diameter of follicle has always been less than 8 mm) group. Twelve cows were selected as the normal estrus group (E, *n* = 6) and inactive ovary group (IO, *n* = 6), and other dairy cows were removed. Twelve experimental dairy cows were slaughtered to collect follicular fluid samples for metabolomics analysis.

All blood and follicular fluid samples from the two groups of IO and E were collected in strict accordance with the guideline on the Protection and Utilization of Experimental Animals in China, and all procedures were approved by the Animal Protection and Utilization Committee of Heilongjiang Bayi Agricultural University.

### 2.2. Sample Pretreatment

Twelve ×100 *μ*l aliquot follicular fluid samples were taken out from -80°C and thawed slowly at 4°C. To each sample, 400 *μ*l methanol and acetonitrile solution (1 : 1, vol/vol) was added to precipitate proteins. All the samples keep in eddy current at -40°C for 60 min to promote precipitation. Then, they were centrifuged at 12000 rpm for 15 min, and the supernatant collected. Both groups (E and IO) of treated samples were mixed in the same amount, and quality control (QC) samples were prepared to evaluate system stability over the entire experiment before testing. After sample pretreatment, samples were sent to Shanghai Applied Protein Technology Co., Ltd. for liquid chromatography-tandem mass spectrometry (LC/MS) analysis.

### 2.3. Metabolite Detection Analysis

LC-MS/MS analysis was performed using an UHPLC system (Vanquish, Thermo Fisher Scientific) with a UPLC BEH Amide column (2.1mm × 100mm, 1.7 *μ*m) coupled to Q Exactive HFX mass spectrometer (Orbitrap MS, Thermo). Pretreated samples were separated by ultra-high-performance liquid chromatography (UHPLC), combined with a hydrop interaction liquid chromatography (HILIC) column (2.1mm × 100mm, 1.7 *μ*m; Waters, Milford, Massachusetts, USA). The column temperature was 35°C, and the injection volume was 3 *μ*l. The mobile phase consisted of A (water + 25mmol/Lofammoniumacetate + 25mmol/Lofammonia) and B (acetonitrile). The elution gradient procedure was as follows: 0–0.5 min; 95% B; 0.5–7 min; 95%–65% B; 7–8 min; 65%–40% B; 8–9 min; 40% B; 9.1–12 min; 95% B. The mobile phase flow rate was 0.5 ml/min, and the automatic sampler was chilled to 4°C. Samples were analyzed using a mass spectrometer (Orbitrap MS, Thermo) that collected primary and secondary mass spectrometry data. Parameters were set as follows: ion source temperature, 320°C; MS resolution, 60000; MS/MS resolution, 7500; collision energy, 35 ± 15 eV; ionization voltage, ± 3.5 kV; scan accumulation time, 0.2 s/spectrum; and precursor ion scan accumulation time, 0.05 s/spectrum. Tandem mass spectrometry data acquisition adopted information-dependent and high-sensitivity acquisition.

### 2.4. Data Processing and Statistical Analysis

The raw data were converted to the mzXML format using ProteoWizard and processed with an in-house program, which was developed using R and based on XCMS, for peak detection, extraction, alignment, and integration. Then, an in-house MS2 database (BiotreeDB) was applied in metabolite annotation. After the data had been preprocessed by pareto-scaling, pattern recognition was performed using the SIMCA-P software (version 15.0.2, Umetrics, Umea, Sweden), consisting of unsupervised principal component analysis (PCA) and supervised orthogonal partial least square discriminant analysis (OPLS-DA). The variable importance in projection (VIP) was performed using the plsr function provided by the R pls package [13] to identify the differential metabolites between the groups.

The data were processed by logarithmic (LOG) conversion and centralized (CTR) formatting, and automatic modeling and analyses were conducted. The results were processed by orthogonal partial least square discriminant analysis (OPLS-DA). OPLS-DA models were tested based on the interpretation of variations in *Y* (*R*^2^*Y*) and forecast ability, based on the model (*Q*^2^) in cross validation (7-fold cross validation). When *R*^2^*Y* ≤ 1 and *Q*^2^ ≥ 0.4, the models were stable and reliable [14]. In addition, the *Q*^2^intercept < 0.05 of the replacement test was used to verify no overfitting [15]. We also conducted univariate analyses, including Student's *t*-tests and fold change analyses. Finally, significantly differential metabolites were screened using variable importance in projection (VIP) scores (VIP > 1) obtained from the OPLS-DA model and *P* values (*P* < 0.05) obtained from *t*-tests.

As shown in Table 1, the independent samples *t*-test was used to compare clinical data (age, parity, BCS, MY, BHBA, NEFA, and Glu) of cows between the E and IO groups using IBM SPSS Statistics for Windows version 19.0 (IBM Corp., USA). *P* < 0.05 and *P* < 0.01 were considered significant and very significant, respectively.

### 2.5. Metabolite Identification and Pathway Analyses

The differential metabolites were identified by searching an in-house standard MS/MS library, the Madison Metabolomics Consortium Database (MMCD), and Human Metabolome Database (HMDB) using MS/MS spectra or exact mass data. The in-house library contains MS/MS spectra of approximately twenty thousand compounds. The MS/MS spectra matching score was calculated using the dot-product algorithm, and the score cutoff was set as 0.8 [16].

We screened the differential metabolites in follicular fluid using untargeted metabolomics and then the differential metabolites selected by multivariate statistical analysis and univariate analysis with the Kyoto Encyclopedia of Genes and Genomes (KEGG) pathway database to sort out all the pathways corresponding to the differential metabolite mapping and performed topological analysis and enrichment analysis of the pathways where the differential metabolites were located to find the key metabolic pathways with the highest correlation with the different metabolites. We subsequently mapped the network metabolism of the differential metabolites to better demonstrate the relationship between the differential metabolites in the screened follicular fluid.

## 3. Results

### 3.1. LC-MS Clinical Data

When compared with the estrus group, BHBA and NEFA levels in blood and milk from the inactive ovary group were significantly higher (*P* < 0.05), and Glu levels in blood and milk from the inactive ovary group were significantly lower (*P* < 0.05), but no significant differences were observed for the age, parity, and BCS (Table 1).

### 3.2. Metabolic Profiles in Follicular Fluids and Data Analysis

**Figure 1:**
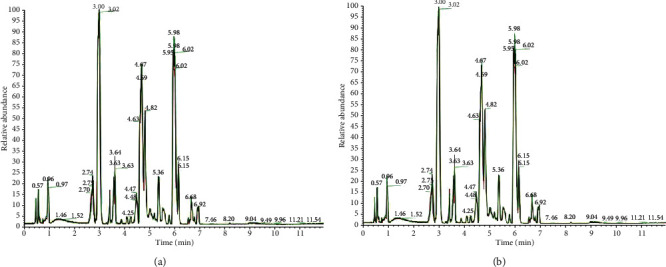
The TIC (total ion count) of the QC (quality control) samples from the IO (inactive ovary cows) and E (estrus cows) groups by liquid chromatography-mass spectrometry (LC-MS): (a) ESI+ (positive ion of electrospray ionization) mode; (b) ESI- (negative ion of electrospray ionization) mode.

We compared total ion chromatograms (TIC) from 12 QC samples in the positive or negative ion mode, including retention times (RT), peak intensity, and resolution. The base peak chromatogram (BPC) of the QC samples displayed good reproducibility, indicating good repeatability and stability. As shown (Figures 1(a) and 1(b)), the BPC peak shape of the base peak chromatogram was complete, and adjacent peaks were well separated from each other. This indicated that chromatographic and mass spectrometric conditions were suitable for sample identification.

**Figure 2:**
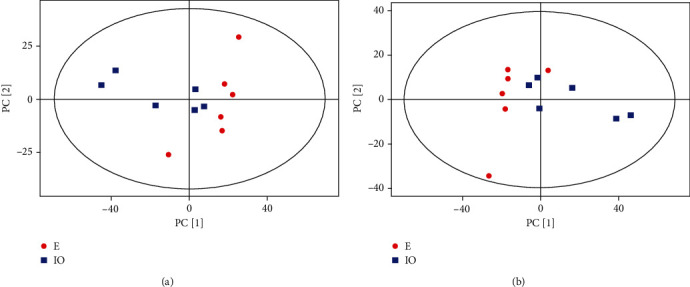
(a) Principal component analysis score plot for the inactive ovaries (IO) and estrus (E) dairy cow follicular fluid samples analyzed in the positive ion mode. (b) Principal component analysis score plot for the inactive ovary (IO) and estrus (E) dairy cow follicular fluid samples analyzed in the negative ion mode. PC [1]: first principal component; PC [2]: second principal component.

**Figure 3:**
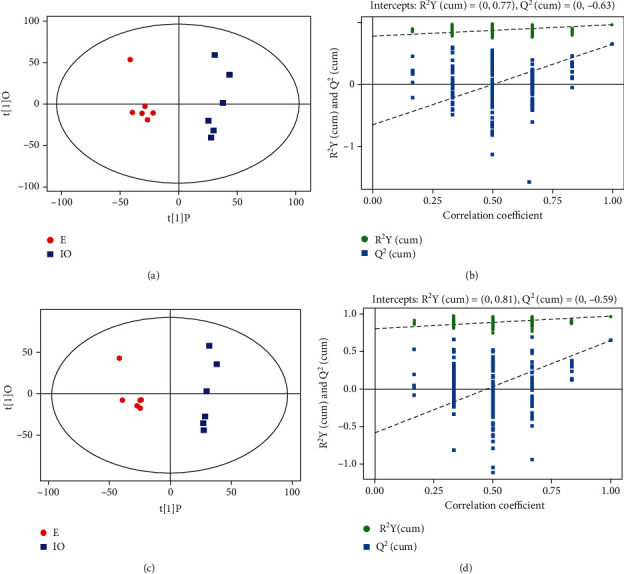
(a, b) Orthogonal partial least square discriminant analysis (OPLS-DA) of scores and permutation test plots for the inactive ovary (IO) and estrus (e) dairy cow follicular fluid samples analyzed in the negative ion mode. (c, d) Orthogonal partial least square discriminant analysis (OPLS-DA) of scores and permutation test plots for the inactive ovary (IO) and estrus (E) dairy cow follicular fluid samples analyzed in the positive ion mode. PC [1]: first principal component; PC [2]: second orthogonal component. The intercept limit of *Q*^2^, calculated by regression line, is the plot of *Q*^2^ from permutation test in the OPLS-DA model.

PCA indicated an overlap between the estrus group and the inactive ovary group, in positive and negative ion mode (Figures 2(a) and 2(b)), which was located within the 95% confidence interval. We used the OPLS-DA supervision model to evaluate metabolite differences between E and IO groups. In the positive ion mode of the OPLS-DA model, *R*^2^*Y* = 0.81 and *Q*^2^ = 0.59, while in the negative ion mode, *R*^2^*Y* = 0.77 and *Q*^2^ = 0.63. *R*^2^*Y* and *Q*^2^ were > 0.4, suggesting that the model was stable, reliable, and predictive (Figure 3). The intercept between the regression line of *Q*^2^ and the longitudinal axis of the positive and negative ion model was <0. When the replacement retention decreased, the proportion of *Y* variables increased gradually, and *Q*^2^ decreased gradually, indicting good model robustness with no overfitting phenomena.

### 3.3. Differential Metabolite Analysis

**Figure 4:**
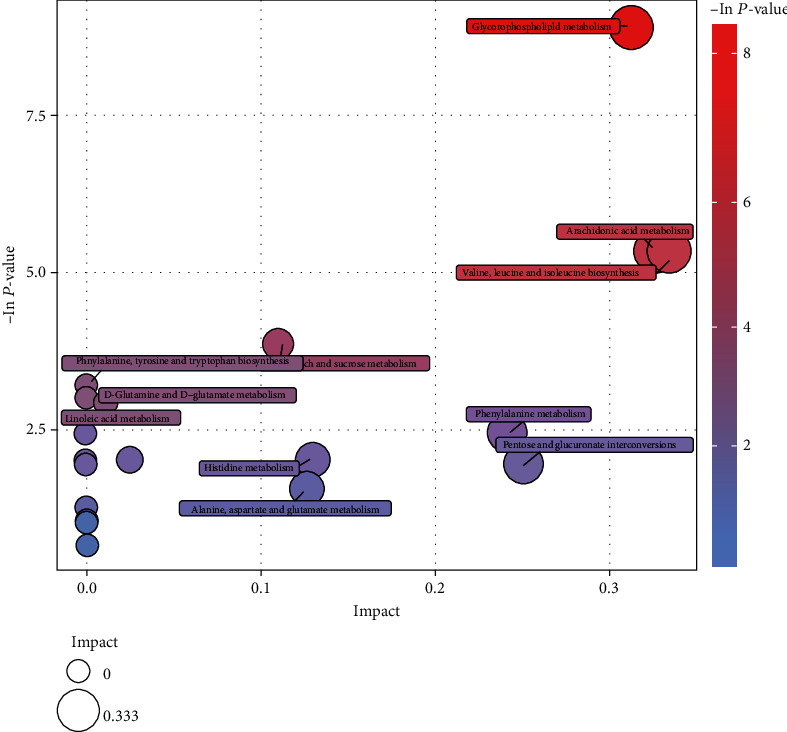
Metabolite pathway analysis of differential follicular fluid in ESI+ (positive ion of electrospray ionization) modes between the E (estrus cows) and IO (inactive ovary cows) groups in LC-MS (liquid chromatography and mass spectrometry) analysis. Circles represent metabolic pathways. The sizes of the bubbles are proportional to the impact of each pathway, with color denoting the significance from the highest in red to the lowest in white.

**Figure 5:**
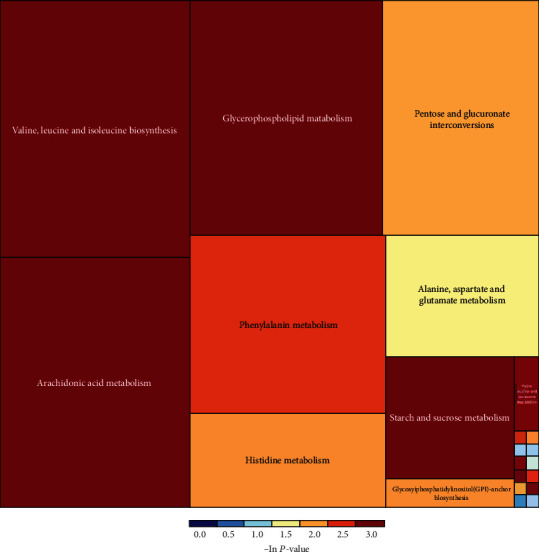
The rectangular tree diagram is used to specifically represent the metabolic pathway related to disease enrichment. The size of the square is the size of the influencing factor of this pathway in topological analysis. The bigger the square is, the greater the influencing factor is. The color is the *P* value (-ln(*P*), that is, the negative natural logarithm) in the enrichment analysis. The smaller of the *P* value, the darker of the color and the more significant of the enrichment degree.

**Figure 6:**
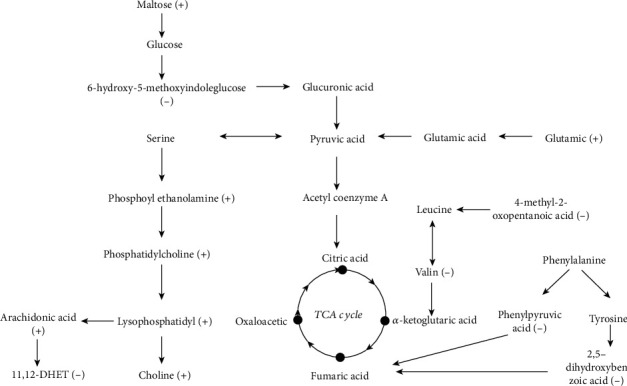
The metabolic pathways of different metabolites from IO (inactive ovary cows) and E (estrus cows) groups are summarized. Compared with group E, the IO group showed a downward and upward adjustment. The pathways for differential metabolites come from the “Encyclopedia of Kyoto Genes and Genomes” database (http://www.kegg.jp) and MetaboAnalyst 3.0 web server (http://www.metaboanalyst.ca). (+) indicates higher concentrations of IO (inactive ovary cows) than E (estrus cows) groups, and (-) indicates lower concentrations of IO (inactive ovary cows) than E (estrus cows) groups. TCA: tricarboxylic acid.

A VIPscore > 1 and *P* < 0.05 was set as criteria for differential metabolite screening. In total, 14 differential metabolites were identified, seven were detected in positive ion mode and seven in negative ion mode. In the inactive ovary group, the expression levels of eight metabolites increased, namely, L-glutamine, choline, lysophosphatidylcholine, phosphatidylcholine, phosphatidylethanolamine, arachidonic acid, maltose, and uric acid. In contrast, in the same group, the expression levels of six metabolites were decreased: valine, 4-methyl 2-oxo-2-valerate, benzene pyruvic acid, 2,5-dihydroxybenzoic acid, (5Z, 8Z, 14Z) -11,12-dihydroxy eicosapentaenoic acid -5,8, and 14-trienoic acid, and 6-hydroxy-5-methoxyindole glucuronic acid (Table 2). These differentially expressed metabolites were analyzed by KEGG and pathways elucidated and identified. They included glycerol phospholipid metabolism, arachidonic acid metabolism, biosynthesis of valine, leucine and isoleucine, and phenylalanine metabolism (Figure 4). Metabolic pathways related to disease are shown in a rectangular tree (Figure 5). These differential metabolites were also involved in protein metabolism, fat metabolism, amino acid metabolism, and nucleotide metabolism. We also analyzed the expression levels of these metabolites in the different metabolic pathways (Figure 6).

## 4. Discussion

Follicular fluids are mainly derived from blood osmosis, hormones, and cytokines secreted by granulosa and membrane cells [17]. Therefore, compositional changes in follicular fluids reflect not only the functional state of the follicle itself, but also the developmental potential and oocyte quality. In this study, we identified 14 differential metabolites in follicular fluids between inactive ovaries and healthy cows, using untargeted metabolomics. These molecules were primarily connected to glycerol phospholipid metabolism, arachidonic acid metabolism, valine, leucine and isoleucine biosynthesis, and phenylalanine metabolism.

### 4.1. Changes in Amino Acid Metabolism

Valine is a branched chain and sugar-producing amino acid, which regulates blood sugars, provides energy for the body, and can be converted to 2-oxo-2-valerate 4-methyl ester via enzymatic processes [18]. 2-Oxo-2-valerate 4-methyl esters generate leucine via branched-chain amino acid transaminase [19]. Leucine is also a branched-chain amino acid. It enters the tricarboxylic acid cycle via enzymatic processes and provides energy for the body [20]. In our research, valine and 4-methyl 2-oxo-2-valerate levels were simultaneously decreased, indicating that valine and leucine in follicular fluid of dairy cows with negative energy balance were consumed for supply energy.

L-Glutamine produces *β*-D-fructose-6-phosphate in the glycolysis/gluconeogenesis pathway, via glutamine-fructose-6-phosphate transaminase and glucosamine-6-phosphate deaminase, or it is converted via glutamate synthetase to glutamate to provide energy for cells [21, 22]. Other studies have shown that glutamine is a metabolic precursor for hexosamine synthesis, which promotes cumulus cell proliferation [23]. L-Glutamine upregulation in inactive ovary cows suggests that glutamine may accumulate in follicular fluids. Then, entry into the glucose metabolic pathway and participation in hexosamine synthesis are blocked. The utilization of L-glutamine in follicular fluid of dairy cows with negative energy balance was insufficient.

In addition, upregulated glutamine may also inhibit glutathione synthesis, because glutamine and cysteine utilize the same transport system [24]. Excessive glutamine can competitively inhibit cysteine uptake, and importantly, cysteine acid is a key factor in glutathione synthesis [8]. Glutathione has antioxidative and free radical-scavenging roles. The concentration of glutathione in oocytes can reflect the degree of oocyte maturation [25]. Therefore, it is speculated that upregulated L-glutamine expression affects glutathione levels in cells, which in turn affects follicular cellular development. L-Glutamine can also enter the purine metabolism pathway via amide phosphoribosyltransferase, which is consistent with our observation of upregulated uric acid in follicular fluid.

Phenylpyruvic acid is produced by phenylalanine via L-amino acid oxidase, and tyrosine is formed after transformation, to participate in glucose and lipid metabolism [26]. 2,5-Dihydroxybenzoic acid is synthesized by the phenylalanine. Phenylpyruvic acid can enzymatically form fumarate to enter into the tricarboxylic acid cycle. 2,5-Dihydroxybenzoic acid can produce fumarate or pyruvate under the action of enzyme, which is involved in metabolic energy supply [27]. In this study, phenylpyruvate and 2-dihydroxybenzoic acid expression in experimental animals were downregulated, while phenylalanine and intermediate tyrosine expression were normal, suggesting that the former metabolites were consumed in the follicles of inactive ovary dairy cows.

### 4.2. Changes in Lipid Metabolism

Arachidonic acid is the most common fatty acid in follicular fluids [28]. In our study, arachidonic acid levels in inactive ovary cows were increased, consistent with Forde and Whelan [29]. Another study suggested that high levels of arachidonic acid can induce granulosa cell death in the ovaries and inhibit estrogen synthesis of these cells [30]. In our work, arachidonic acid levels in inactive ovary cows were increased, while 12-dihydroxy eicosapentaenoic acid and 5-dihydroxy eicosapentaenoic acid levels were decreased, suggesting that their catabolism decreased, and a large amount of accumulation in follicular fluid led to an increase in arachidonic acid content, which affected the synthesis of granulosa cells and their steroid hormones and then affected the development of follicles.

Lysophosphatidylcholine, phosphatidylcholine, phosphatidylethanolamine, and choline expression were upregulated, suggesting that glycerol phospholipid metabolism in the follicular fluids of experimental animals was enhanced. Serine is enzymatically converted to phosphatidylserine, which is metabolized by phosphatidylserine decarboxylase to form phosphatidylethanolamine. After a further series of enzymatic reactions, acetyl coenzyme A is formed, which enters the tricarboxylic acid cycle. Or under the action of phospholipase C and diacylglycerol choline phosphate transferase to form phosphatidylcholine, phosphatidylcholine under the action of lecithin cholesterol acyltransferase to form lysophosphatidylcholine finally converted to choline. Choline is a component of biofilms and precursor of the neurotransmitter, acetylcholine [31]. The molecule has important physiological functions, i.e., ensuring information transmission, regulating apoptosis, promoting fat metabolism, and eliminating cholesterol from serum [32]. Choline expression was upregulated in inactive ovary cows; we believe this may be due to an enhancement of glycerol phospholipid metabolism in dairy cows with inactive ovaries, or alternatively, choline cannot participate in cell membrane synthesis due to the cessation of follicular development. Phosphatidylcholine and phosphatidylethanolamine are both antioxidants, and their upregulated expression is related to enhanced glycerol phospholipid metabolism and antioxidation [33]. Lysophosphatidylcholine also activates the PPAR*γ* pathway [34], with some studies showing that PPAR*γ* activation regulates cholesterol 7*α*-monooxygenase (CYP7A1) through the PPAR signal pathway, thus affecting steroid production [35]. In cultured human granulosa cells, the PPARG activator, troglitazone inhibited gonadotropin-stimulated progesterone production [36]. It has been reported that PPARG activation inhibits the growth of dominant follicles in bovine and granulosa cells, regulates the production of steroids, and causes bovine follicular atresia [37]. The enhancement of glycerol phospholipid metabolism in follicular fluids of dairy cows with inactive ovaries, especially lysophosphatidylcholine in follicular development, warrants further study.

### 4.3. Changes in Glucose Metabolism

In this experiment, the content of maltose in follicular fluid increased and the content of 6-hydroxy-5-methoxyindole glucuronic acid decreased in inactive ovary dairy cows, suggesting that glycogen decomposition increased and supplied energy for follicular development [38]. 6-Hydroxy-5-methoxyindole glucuronic acid can be converted to glucuronic acid and then enzymatically converted to pyruvate to provide energy, or it can be converted to ascorbic acid (vitamin C) in the presence of glucuronidase [39]. Vitamin C is also a highly effective antioxidant that scavenges free radicals [40]. Thus, the downregulation of 6-hydroxy-5-methoxyindole glucuronic acid expression, with concomitant changes in vitamin C expression, indicated that the main metabolic direction is producing pyruvate through *β*-glucuronidase pathway for energy supply.

In this study, 14 metabolites in follicular fluids of dairy cows with inactive ovaries were identified using untargeted metabolomics (LC-MS/MS) to explore their potential role in the development of inactive ovaries, but it still needs further research for enlarging the number of the experiment cows and verifying the metabolite and related enzymes of the metabolic pathways in the future.

## 5. Conclusion

In this study, a metabolomics-based analysis using a UHPLC-TOF/MS approach was used to study metabolic changes in follicular fluids from dairy cows with inactive ovaries. We identified 14 metabolites that were differentially expressed at 45-60 days after delivery. Additionally, we observed differences in glycerol phospholipid metabolism, arachidonic acid metabolism, valine, leucine and isoleucine biosynthesis, and phenylalanine metabolism through a map of the interaction among differential metabolites in follicular fluid of dairy cows with inactive ovaries. These differential metabolites indicated the pathway of negative energy balance impairing follicular development in dairy cows.

## Figures and Tables

**Table 1:** Clinical data in two groups of dairy cows.

Parameters	E (*n* = 6)	IO (*n* = 6)
Age	3.37 ± 0.54	3.13 ± 0.92
Parity	2.33 ± 0.52	2.00 ± 0.89
BCS	2.92 ± 0.49	2.67 ± 0.20
MY (kg/d)	38.48 ± 3.47	44.00 ± 4.17^∗^
BHBA (mmol/L)	0.78 ± 0.37	1.37 ± 0.49^∗^
NEFA (mmol/L)	0.44 ± 0.09	0.73 ± 0.25^∗^
Glu (mmol/L)	3.63 ± 0.35	2.99 ± 0.20^∗^

E: estrus cows; IO: inactive ovary cows; BCS: body condition score; MY: milk yield per day; Glu: glucose; NEFA: nonesterified fatty acid; BHBA: *β*-Hydroxybutyric acid. ^∗^*P* < 0.05; ^∗∗^*P* < 0.01.

**Table 2:** Differential follicular fluid metabolites of E and IO cows screened by LC-MS.

ID	Metabolites	RT(s)	VIP value	*P* value	Change trend	Mode
1	Valine	302.69	1.92	0.0001	↓	ESI-
2	L-Glutamine	406.31	1.47	0.0337	↑	ESI+
3	4-Methyl 2-oxo-2-valerate	57.88	1.83	0.0018	↓	ESI-
4	Benzene pyruvic acid	92.35	1.41	0.0330	↓	ESI-
5	2,5-Dihydroxybenzoic acid	61.34	1.44	0.0233	↓	ESI-
6	Choline	280.86	1.70	0.0100	↑	ESI+
7	Lysophosphatidylcholine	211.14	1.85	0.0069	↑	ESI+
8	Phosphatidylcholine	36.10	1.13	0.0403	↑	ESI+
9	Phosphatidylethanolamine	58.74	1.68	0.0153	↑	ESI+
10	Arachidonic acid	46.47	1.45	0.0435	↑	ESI-
11	(5Z, 8Z, 14Z) -11,12-dihydroxy eicosapentaenoic acid -5,8, and 14-trienoic acid	78.28	1.66	0.0098	↓	ESI-
12	Maltose	416.44	1.33	0.0276	↑	ESI-
13	6-Hydroxy-5-methoxyindole glucuronic acid	313.81	1.59	0.0043	↓	ESI+
14	Uric acid	322.52	1.73	0.0202	↑	ESI+

The change trend indicates concentration in the IO group (inactive ovary cows, *n* = 6) higher or lower than that in the E group (estrus cows, *n* = 6). RT: retention time; s: second; VIP: variable importance in projection.

## Data Availability

The datasets used and/or analyzed during the current study are available from the corresponding author on reasonable request.

## References

[1] Walsh S., Williams E., Evans A. (2011). A review of the causes of poor fertility in high milk producing dairy cows. *Animal Reproduction Science*.

[2] Block S. S., Butler W. R., Ehrhardt R. A., Bell A. W., van Amburgh M., Boisclair Y. R. (2001). Decreased concentration of plasma leptin in periparturient dairy cows is caused by negative energy balance. *Journal of Endocrinology*.

[3] Esposito G., Irons P. C., Webb E. C., Chapwanya A. (2014). Interactions between negative energy balance, metabolic diseases, uterine health and immune response in transition dairy cows. *Animal reproduction science*.

[4] Shook G. E. (2006). Major advances in determining appropriate selection goals. *Journal of dairy science*.

[5] Giordano J., Wiltbank M., Guenther J. (2012). Increased fertility in lactating dairy cows resynchronized with Double-Ovsynch compared with Ovsynch initiated 32 D after timed artificial insemination. *Journal of Dairy Science*.

[6] Bisinotto R., Ribeiro E., Lima F. (2013). Targeted progesterone supplementation improves fertility in lactating dairy cows without a corpus luteum at the initiation of the timed artificial insemination protocol. *Journal of Dairy Science*.

[7] Thatcher W. W., Moreira F., Santos J. E. P. (2001). Effects of hormonal treatments on reproductive performance and embryo production. *Theriogenology*.

[8] Mcdougall S. (2006). Reproduction performance and management of dairy cattle. *Journal of Reproduction and Development*.

[9] Beam S. W., Butler W. R. (1998). Energy balance, metabolic hormones, and early postpartum follicular development in dairy cows fed prilled lipid. *Journal of Dairy Science*.

[10] Butler W. (2003). Energy balance relationships with follicular development, ovulation and fertility in postpartum dairy cows. *Livestock Production Science*.

[11] Nicholson J. K., Lindon J. C., Holmes E. (1999). Metabonomics: understanding the metabolic responses of living systems to patho-physiological stimuli via multivariate statistical analysis of biological NMR spectroscopic data. *Xenobiotica*.

[12] Patti G. J., Yanes O., Siuzdak G. (2012). Innovation: metabolomics: the apogee of the omics trilogy. *Nature reviews Molecular cell biology*.

[13] Mevik B. H., Wehrens R. (2007). The pls package: principal component and partial least squares regression in R. *Journal of Statistical Software*.

[14] Westerhuis J. A., Hoefsloot H. C., Smit S. (2008). Assessment of PLSDA cross validation. *Metabolomics*.

[15] Liu H., Tayyari F., Khoo C., Gu L. (2015). A ^1^H NMR-based approach to investigate metabolomic differences in the plasma and urine of young women after cranberry juice or apple juice consumption. *Journal of Functional Foods*.

[16] Stein S. E., Scott D. R. (1994). Optimization and testing of mass spectral library search algorithms for compound identification. *Journal of the American Society for Mass Spectrometry*.

[17] Guzmán C., Hernández-Bello R., Morales-Montor J. (2010). Regulation of steroidogenesis in reproductive, adrenal and neural tissues by cytokines. *Bello*.

[18] Summers M. C., McGinnis L. K., Lawitts J. A., Biggers J. D. (2005). Mouse embryo development following IVF in media containing either l-glutamine or glycyl-l-glutamine. *Human Reproduction*.

[19] Li S., Qi X., Huang B. (2016). Synthesis of 7-hydroxy-4-methylcoumarin via the Pechmann reaction with PVP-supported phosphotungstic acid catalyst. *Catalysis Today*.

[20] Chen L., Wert S. E., Hendrix E. M., Russell P. T., Cannon M., Larsen W. J. (1990). Hyaluronic acid synthesis and gap junction endocytosis are necessary for normal expansion of the cumulus mass. *Molecular Reproduction and Development*.

[21] Sutton-McDowall M. L., Gilchrist R. B., Thompson J. G. (2010). The pivotal role of glucose metabolism in determining oocyte developmental competence. *Reproduction*.

[22] Furnus C. C., de Matos D. G., Moses D. F. (1998). Cumulus expansion during in vitro maturation of bovine oocytes: relationship with intracellular glutathione level and its role on subsequent embryo development. *Molecular Reproduction and Development*.

[23] de Matos D. G., Furnus C. C. (2000). The importance of having high glutathione (GSH) level after bovine in vitro maturation on embryo development: effect of *β*-mercaptoethanol, cysteine and cystine. *Theriogenology*.

[24] Tompkins S. C., Sheldon R. D., Rauckhorst A. J. (2019). Disrupting mitochondrial pyruvate uptake directs glutamine into the TCA cycle away from glutathione synthesis and impairs hepatocellular tumorigenesis. *Cell Reports*.

[25] Grochowska R., Sørensen P., Zwierzchowski L., Snochowski M., Løvendahl P. (2001). Genetic variation in stimulated GH release and in IGF-I of young dairy cattle and their associations with the leucine/valine polymorphism in the GH gene. *Journal of Animal Science*.

[26] Lemmon M. A., Schlessinger J. (2010). Cell signaling by receptor tyrosine kinases. *Cell*.

[27] Bossaert P., Leroy J. L. M. R., de Vliegher S., Opsomer G. (2008). Interrelations between glucose- induced insulin response, metabolic indicators, and time of first ovulation in high-yielding dairy cows. *Journal of Dairy Science*.

[28] Liu J., Brown R. E. (2011). Immunohistochemical expressions of fatty acid synthase and phosphorylated c-Met in thyroid carcinomas of follicular origin. *International Journal of Clinical &Experimental Pathology*.

[29] Forde N., O'Gorman A., Whelan H. (2015). Lactation-induced changes in metabolic status and follicular-fluid metabolomic profile in postpartum dairy cows. *Reproduction, Fertility, and Development*.

[30] Hjort Ipsen J., Karlström G., Mourtisen O. G., Wennerström H., Zuckermann M. J. (1987). Phase equilibria in the phosphatidylcholine-cholesterol system. *Biochimica et Biophysica Acta*.

[31] Gibb A. J. (2017). Choline and acetylcholine: what a difference an acetate makes. *The Journal of Physiology*.

[32] Komar C. M., Braissant O., Wahli W., Curry Jr T. E. (2001). Expression and localization of PPARs in the rat ovary during follicular development and the periovulatory period. *Endocrinology*.

[33] Schneider W. J., Vance D. E. (1978). Effect of choline deficiency on the enzymes that synthesize phosphatidylcholine and phosphatidylethanolamine in rat liver. *European Journal of Biochemistry*.

[34] Wang X. J., Dyson M. T., Jo Y., Eubank D. W., Stocco D. M. (2003). Involvement of 5-lipoxygenase metabolites of arachidonic acid in cyclic AMP-stimulated steroidogenesis and steroidogenic acute regulatory protein gene expression. *Journal of Steroid Biochemistry and Molecular Biology*.

[35] Gupta S., Stravitz R. T., Dent P., Hylemon P. B. (2001). Down-regulation of cholesterol 7*α*-hydroxylase (*CYP7A1*) gene expression by bile acids in primary rat hepatocytes is mediated by the c-Jun N-terminal kinase pathway. *Journal of Biological Chemistry*.

[36] van der Greef J., Smilde A. K. (2005). Symbiosis of chemometrics and metabolomics: past, present, and future. *Journal of Chemometrics*.

[37] Cetica P., Pintos L., Dalvit G., Beconi M. (2002). Activity of key enzymes involved in glucose and triglyceride catabolism during bovine oocyte maturation in vitro. *Reproduction*.

[38] Pangas S. A., Saudye H., Shea L. D., Woodruff T. K. (2003). Novel approach for the three-dimensional culture of granulosa cell-oocyte complexes. *Tissue Engineering*.

[39] Monteiro M., Carvalho M., Bastos M. L., Guedes de Pinho P. (2013). Metabolomics analysis for biomarker discovery: advances and challenges. *Current medicinal chemistry*.

[40] Bender K., Walsh S., Evans A., Fair T., Brennan L. (2010). Metabolite concentrations in follicular fluid may explain differences in fertility between heifers and lactating cows. *Reproduction*.

